# The intersection of turn-taking and repair: the timing of other-initiations of repair in conversation

**DOI:** 10.3389/fpsyg.2015.00250

**Published:** 2015-03-12

**Authors:** Kobin H. Kendrick

**Affiliations:** Language and Cognition Department, Max Planck Institute for Psycholinguistics, NijmegenNetherlands

**Keywords:** conversation analysis, turn-taking, timing, delay, other-initiated repair, self-repair, preference

## Abstract

The transitions between turns at talk in conversation tend to occur quickly, with only a slight gap of ∼100–300 ms between them. This estimate of central tendency, however, hides a wealth of complex variation, as a number of factors, such as the type of turns involved, have been shown to influence the timing of turn transitions. This article considers one specific type of turn that does not conform to the statistical trend, namely turns that deal with troubles of speaking, hearing, and understanding, known as other-initiations of repair (OIR). The results of a quantitative analysis of 169 OIRs in face-to-face conversation reveal that the most frequent cases occur after gaps of ∼700 ms. Furthermore, OIRs that locate a source of trouble in a prior turn specifically tend to occur after shorter gaps than those that do not, and those that correct errors in a prior turn, while rare, tend to occur without delay. An analysis of the transitions before OIRs, using methods of conversation analysis, suggests that speakers use the extra time (i) to search for a late recognition of the problematic turn, (ii) to provide an opportunity for the speaker of the problematic turn to resolve the trouble independently, and (iii) to produce visual signals, such as facial gestures. In light of these results, it is argued that OIRs take priority over other turns at talk in conversation and therefore are not subject to the same rules and constraints that motivate fast turn transitions in general.

## Introduction

In conversation opportunities to participate are organized by a system of turn-taking (Sacks et al., 1974). The rules and constraints of the turn-taking system conspire to minimize the duration of transitions between turns. But not all transitions are in fact minimal. The transitions before turns that deal with troubles of speaking, hearing, and understanding, known as other-initiations of repair (OIRs; e.g., “what?," “who?," “what’d you mean?"), have been reported by Schegloff et al. (1977) to be longer than those before other turns. How much longer, however, remains an open question. The first goal of this investigation is therefore to verify and refine this observation though a quantitative analysis of the timing of OIR, using responses to polar question as a point of comparison. Schegloff et al. (1977) argue that next speakers withhold OIRs to provide an opportunity for current speakers to resolve the trouble via self-repair. Whether this exhausts the possible explanations for delay before OIR is unclear, however. The second goal of the investigation is therefore to look inside the transition spaces before OIRs, using conversation-analytic methods to discover and describe what speakers use them to accomplish. As background to this, I begin with a discussion of the timing of turn-taking in general and the timing of OIR more specifically.

### The Timing of Turn-Taking

Previous research on the timing of turn-taking has shown that the transitions between turns in conversation most frequently occur with only minimal gaps and overlaps. First documented systematically in a series of meticulous conversation-analytic studies (Jefferson, 1973, 1983a,b, 1984, 1986; Sacks et al., 1974), the timing of transitions between turns has subsequently been investigated primarily through quantitative methods. In general, quantitative studies have taken one of two approaches, either examining all transitions within a corpus, irrespective of turn type (e.g., Wilson and Zimmerman, 1986; Heldner and Edlund, 2010), or analyzing just one type of transition, that between questions and answers (e.g., Stivers et al., 2009; Kendrick and Torreira, 2014). A comparison between the timing of transitions between questions and answer and that of a random sample of transitions in a corpus of Dutch conversation revealed no statistically significant difference (Stivers et al., 2009). This suggests that the timing of question–answer sequences can be used as a proxy for a typical turn transition in conversation. The results of these studies generally converge, indicating that the most frequent transitions between turns occur with a slight gap (cf. Jefferson, 1984, p. 18), on the order of 100–300 ms.

This estimate of central tendency has clear implications for psycholinguistic models of turn-taking. As Levinson (2013) points out, psycholinguistic research has shown that speakers require a minimum of 600 ms to plan even a single word (e.g., in a picture-naming task; Indefrey and Levelt, 2004; Indefrey, 2011). Thus the average gap between turns does not provide enough time for a speaker to prepare even a simple next turn. Therefore, Levinson argues, next speakers must anticipate the ends of turns, and begin to plan the next turn well before the current one is complete, in full agreement with arguments by Sacks et al. (1974) and Schegloff (1987).

But an estimate of central tendency is by definition a simplification, a single value that ideally represents a more complex distribution. Research has also examined sources of complex and systematic variation in the timing of turn-taking, especially in question–answer sequences. The language and culture of the speakers, the deployment of gaze, and the type and modality of the response all have been shown to influence the timing of responses to polar questions (Stivers et al., 2009). Question type is also relevant; responses to polar questions are generally faster than responses to content questions (Strömbergsson et al., 2013). Studies that explore variation in the timing of turn transitions are important because they reminds us of the diversity of turn types and hence turn transitions in conversation.

### The Timing of Other-Initiations

At least one type of turn that does not conform to global generalizations about the timing of turn-taking has been identified in the conversation-analytic literature. Turns that deal with troubles of hearing or understanding prior turns (e.g., “what?,” “who?,” “what’d you mean?”), referred to as OIRs, have been reported to be systematically withheld (Schegloff et al., 1977). The transition between the turn that contains the trouble (e.g., an error or a word the next speaker does not understand), referred to as the trouble-source turn, and the other-initiation of repair has been observed to be longer than other turn transitions (Schegloff et al., 1977; Robinson, 2006).

Based on a systematic qualitative analysis, Schegloff et al. (1977, p. 374) observed that OIRs “regularly are withheld” by speakers and therefore “occur after a slight gap.” Although the report includes numerous cases of OIRs that occur after a slight gap (and many that do not), it leaves basic questions unanswered, such as the frequency with which speakers withhold OIRs and the durations of the gaps that precede them.

A second report of the phenomenon, made in passing by Robinson (2006, p. 153), contains more detailed information. Based on 32 cases of OIR in telephone conversations, Robinson observed that the median delay was between 0.1 and 0.2 s. This finding is not conclusive, however, for two reasons. The first concerns the method of timing. Rather than measure the duration of gaps and pauses objectively (e.g., using a computer), conversation analysts typically employ a relative method of timing, one that reflects the analyst’s perception of time (Hepburn and Bolden, 2013), a method that has been shown to overestimate objectively measured time systematically (Roberts and Robinson, 2004; Kendrick and Torreira, 2014). The second issue is the lack of an explicit comparison between the timing of OIRs and the timing of other turns in the same conversations. To conclude that OIRs are delayed systematically, one must establish not only that gaps before them are long, but more importantly that they are *longer* than gaps before other turns. Thus while Robinson’s finding supports the claim by Schegloff et al. (1977), the frequency with which speakers do or do not withhold OIRs and the precise timing of the gaps that precede them remain open questions.

### The Practices of Other-Initiation

The observation of systematic variation in the timing of turn types (e.g., responses to questions, noted above) points to further questions. Is variation in the timing of OIRs also systematic? Do different types of OIRs, like different types of responses to questions, tend to occur after relatively shorter or longer transitions?

The inventory of OIR practices in English is relatively well described (see Schegloff et al., 1977; Benjamin, 2013; Kitzinger, 2013; Kendrick, in press). A basic distinction is made between OIRs that pinpoint a specific source of trouble in a prior turn and those that do not. Open OIRs such as “what?” or “sorry?” indicate that the speaker has encountered a trouble but do not specify a particular source (Drew, 1997). In contrast, OIRs that repeat all or part of a prior turn (e.g., “they’re what?”) or request category-specific information (e.g., “who?”), among other possibilities, specifically locate the source of the trouble. According to Schegloff et al. (1977), there is a preference for more specific (‘stronger’) over less specific (‘weaker’) OIRs, such that speakers should, for example, use a specific OIR over an open OIR if possible (cf. Svennevig, 2008). This raises the question of whether the timing of open and specific OIRs differs systematically and whether it provides evidence for or against the preference for specificity.

A further distinction is whether an OIR constitutes a correction or not (Schegloff et al., 1977; Jefferson, 1987). An OIR can proffer a replacement of a trouble source as a candidate solution to a trouble of hearing or understanding (e.g., A: “she got so mad.” B: “Pam’s mother?” A: “Mm hm.”). Or it can assert a replacement as correction of an error (e.g., A: “she’s eating the Butterworth diet.” B: “Butterfield.” A: “Butterfield.”). Correction by someone other than the speaker of the error, known as other-correction, has been argued to be a dispreferred alternative to self-correction. Schegloff et al. (1977) observed that other-corrections tended to exhibit special marking and special positioning (e.g., the qualification of epistemic stance or delay within a turn or sequence) that revealed them to be dispreferred actions. But counter-examples to this generalization are not uncommon (see, e.g., Jefferson, 1987, p. 87), and a recent survey of other-correction in Finnish failed to identify special markings of the type that Schegloff et al. described (Haakana and Kurhila, 2009). What, then, of the timing of other-corrections? Are the transitions before other-corrections longer than other turns, as their putative status as dispreferred actions predicts?

### The Motivations for Delay Before Other-Initiations

A final set of questions concerns the motivations for and consequences of delay before OIR. Conversation analysts have argued that the timing of OIRs has a socio-interactional basis. A possible next speaker who encounters a problematic turn withholds an OIR in order to provide the current speaker with an opportunity to resolve the trouble on his or her own (Schegloff et al., 1977). If a next speaker does not understand a question, for example, he or she might not immediately reply with “what’d you mean?” but might first wait for a moment to give the current speaker an opportunity to repair the question independently, using practices of self-initiated repair. In this way, the self-initiation of repair takes precedence over the other-initiation of repair, one aspect of a principle known as the preference for self-correction (Schegloff et al., 1977). The timing of the transition between a trouble-source turn and an other-initiation of repair is thus seen as a locus for the management of basic social relations, between self and other.

But a priori one might propose complementary or alternative explanations for the phenomenon of delay before OIR. The private processes that speakers necessarily engage in to hear and understand a turn at talk occur in real time and, just like other actions, take time to complete. The high frequency of transitions with minimal gaps suggests that these processes very often occur quickly. But might they not take longer under certain conditions, say, when a next speaker has failed to hear, or understand a prior turn? Is the delay before an OIR for the benefit of the speaker of the trouble, to provide an opportunity for self-initiated repair, or is it (also) for the benefit of the recipient of the trouble, to allow a search for and a possible recovery of a hearing and understanding of the turn that may permit the sequence to move forward without repair?

One might also look to the embodied actions of the participants for an explanation. The private processes that participants engage in surely take time to complete, but so too do the physical actions they perform. A withdrawal of gaze from the speaker of a trouble-source turn, a rotation of the head and body to face a trouble-source speaker, a meaningful deployment of facial muscles into a gesture of puzzlement—embodied actions such as these all take time to produce. Through the filter of a text transcript, a silence between turns at talk, whether long, or short, can look like an absence of action. But the long tradition of multimodal conversation analysis (Goodwin, 1980, 1981; Schegloff, 1998; Lerner, 2003; Mondada, 2006, 2007; Rossano, 2012; inter alia) has shown that action does not necessarily end with a turn at talk. What, then, do speakers *do* in the transition spaces before OIR?

### The Current Investigation

The current investigation combines conversation-analytic and quantitative methods to address the following questions about the timing of OIR.

(1) Are the transition spaces before OIRs systematically longer than those before other turn types, such as answers to questions?
(2) Are the transition spaces before some types of OIRs systematically longer than those before other types?
(3) What are the motivations for the expansion of transition spaces before OIRs? That is, what do speakers use the extra time to accomplish?

The investigation uses conversation analysis to identify and analyze OIRs and question–answer sequences, as well as to discover and describe a number of uses that speakers have for the expanded transition spaces before OIRs. The measurements of transition spaces and the comparisons of the distributions are done quantitatively. At the end of the article I return to the questions of the motivations for and consequences of delay before OIRs and consider the relationship between repair and the turn-taking system.

## Materials and Methods

### Data

The data for the investigation came from video-recordings of naturally occurring English conversation between friends and family members engaged in a variety of activities (e.g., chatting, playing games, preparing food, eating dinner). The corpus consisted of 19 recordings, with a total duration of 9 h and 20 min, and included native speakers of English from the U.S., Canada, and the U.K. Informed consent was obtained from all participants.

### Identification of OIR

All cases of OIR were systematically identified in the corpus, using the methods of conversation analysis and drawing on previous research on OIR (Schegloff et al., 1977; Jefferson, 1987; Schegloff, 1992; Robinson, 2006; Egbert et al., 2009; Robinson and Kevoe-Feldman, 2010; Benjamin and Walker, 2013). OIRs were distinguished from formally similar practices that do not initiate repair as an action (see Schegloff, 1997, for examples). It is well known that the practices of OIR can be used to display surprise or ritualized disbelief (Selting, 1988; Wilkinson and Kitzinger, 2006). In the case of repeats of a trouble source, a clear boundary between such cases and those that additionally or alternatively display surprise or disbelief has not yet been identified in the literature. Such cases were therefore included in the investigation. The types of OIRs identified in the corpus are given in **Table 1** along with examples (for a more detailed report of the distribution of OIR in English conversation, see Kendrick, in press). A total of 222 cases of OIR were identified, for an average rate of one every 2.5 min.

**Table 1:** Frequency and proportion of other-initiations of repair in a contiguous next position to trouble-source turn-constructional units (TCUs).

Type	Example	Frequency	Proportion %
Open	*what?, huh?, pardon?, what’s that?,* among others	53	31.0
Interrogative words	*who?, when?, where?,* and *what* with falling intonation	11	6.4
Repeats + interrogative word	A: *A plastic bag if you could.* B: *A what?* A: *Plastic bag.*	19	11.1
Full repeats	A: *And we have things to finish.* B: *We have things to finish?* A: *That we started earlier.*	9	5.3
Partial repeats	A: *We could start a little school together.* B: *Little school?* A: *Yeah, like Angel was gonna do.*	20	11.7
Candidate understandings	A: *Nan’s birthday on Sunday.* B: *Norms?* A: *No, Shirley.*	42	24.6
Corrections	A: *Transforming Investments* B: *Translating Investments. Sorry.* A: *Translating Investments.*	12	7.0
Other	*I don’t know who that person is, who’s the guy,* among others	5	2.9

### Open and Specific OIRs

Other-initiations of repair differ in the specificity with which they locate the source of trouble in the prior talk (Schegloff et al., 1977). Open OIRs indicate that the speaker has encountered trouble with the prior talk but they do not specify a particular source (e.g., “what?”; see Drew, 1997; Benjamin, 2013). In contrast, specific OIRs locate a particular component in the prior talk as the trouble source (e.g., “who?” or “she did what?”). The practices listed **Table 1**, other than those designated as open, were analyzed as specific.

### Candidate Repair Solutions and Other-Corrections

Other-initiations of repair that include possible solutions to the trouble differ in whether the solution is offered as a candidate replacement of the trouble source or asserted as a correction of the trouble source. These two alternatives are illustrated in Extract (1) and Extract (2), respectively.


(1) Virginia
1 Wes:	    ◦Here you go◦
2 Bet:	    (But) she[: got so: ma:d.
3 Vir:	    	    [◦Thank you◦
4 	    (3.2)
5 Mom: ->   Pam’s mother?
6 Bet:	    Mm: hm


(2) RCE09
1 Ben:	    She’s ea(h)ting the Butterwo(h)rth
2	    di[e(h)t.
3 Jam: ->   [Bu(h)tterfie(h)ld.
4	    (0.9)
5 Ben:	    Butterfield.


In Extract (1) the other-initiation of repair “Pam’s mother?” (line 5) is a candidate solution to the speaker’s trouble with the reference “she” (line 2). The candidate is produced with rising intonation, which qualifies the speaker’s epistemic stance. The speaker thereby offers this as a possible, but not definitive, solution to the trouble. In contrast, the other-initiation of repair in Extract (2) is produced with falling intonation and an accent on the third syllable, through which the speaker asserts it as a correction of the trouble source. The cases also differ in the epistemic status (Heritage, 2012) of the speakers. In Extract (1), the mother has only indirect knowledge of the event reported in the trouble-source turn, whereas the speaker in Extract (2) has direct knowledge of the correct name.

This practice of other-correction has also been examined by Jefferson (1987) under the rubric of ‘exposed correction,’ an example of which occurs in Extract (3).

(3) Jefferson (1987:87)
1 Pat:    the Black Muslims are certainly more
2         provocative than the Black Muslims
3         ever were.
4 Jo: →   The Black Panthers.
5 Pat:    The Black Panthers.

To be analyzed as an other-correction, the OIR had to (i) include a possible replacement for the trouble source, (ii) use prosodic resources (an accented syllable and final falling intonation) to assert the replacement as definitive; and (iii) make self-correction (not confirmation) conditionally relevant as a response.

### OIRs in and After Next Position

Although the majority of OIRs occur directly after the turn at talk containing the trouble source, a minority of cases occur after this next-turn position (Schegloff, 2000; Wong, 2000; Bolden, 2009; Benjamin, 2012). A distinction between these two positions is crucial for the present investigation because only OIRs in next position to the turn-constructional unit (TCU; Sacks et al., 1974) that contains the trouble-source result in a transition that consist of a gap or overlap, without intervening talk. The OIR in Extract (4) illustrates this point.

(4) RCE01 09:56
1 Liz:     I don’t- (0.8) I don’t know whether to
2          get a maxi dress for my birthday.
3          (0.5)
4 Liz:     I’ve got one and it[’s k- just
5 Cha: →                      [What’s maxi.=Long?
6 Liz:     Really long, yeah.

The other-initiation “What’s maxi.=Long?” (line 5) does not occur directly after the TCU that contains the trouble source “maxi” (line 2), but rather occurs after the speaker of the trouble source extends her turn with an additional TCU (line 4). The duration of time between the end of the trouble-source TCU and the beginning of the OIR does not constitute a inter-turn gap because it includes intervening talk which affects the timing of the OIR.

All cases of OIR were therefore analyzed for position, following Schegloff (2000), with the requirement that the OIR be in a contiguous next position to the TCU containing the trouble source. The boundaries of TCUs were identified as points of syntactic, intonational, and pragmatic completion (Ford and Thompson, 1996). A total of 51 cases (23% of all cases) did not occur in next position under this definition and were excluded from the analysis of timing.

### Identification of Polar Questions

In addition to OIRs, responses to polar questions were also identified in the corpus for comparison. For each recording, a number of polar questions equal to the number of other-initiations was identified, starting at the beginning of the recording. For example, if 10 other-initiations were identified in a recording, the first 10 polar question sequences were then taken from the same recording. Polar questions were defined functionally to include both syntactic questions (i.e., those with verb inversion) and epistemic questions (i.e., statements about information in the recipient’s epistemic domain, so-called B-event statements; Labov and Fanshel, 1977).

### Measurements and Statistics

The duration of turn transitions were measured from the end of the TCU containing the trouble source to the beginning of the other-initiation of repair, excluding audible in-breaths. Measurements were made manually in ELAN 4.3.3 (Wittenburg et al., 2006) by listening to the audio recording and inspecting the waveform. Two extreme outliers with gap durations greater than 3000 ms were excluded from the quantitative analysis, resulting in a final set of 169 cases. Because the distributions of gap durations were found to deviate substantially from a normal distribution (with skewness and kurtosis values of more than twice their respective standard errors), non-parametric significance tests were used. All statistical tests were performed in R 2.14.0 (R Development Core Team, 2013) with the wilcox_test() function in the coin package (Hothorn et al., 2006).

## Results

### The Timing of Other-Initiations of Repair

The timing of an other-initiation of repair is the duration of the transition space, measured in milliseconds, between the end of the trouble-source TCU and beginning of the OIR (see Materials and Methods). In Extract (5), the OIR at line 4 occurs after a gap of 514 ms. Hereafter the transition spaces before OIRs are reported in milliseconds in all transcripts, whereas others are given as 10ths of seconds, the standard convention in conversation analysis.

(5) Virginia
1 Bet:    They said that Phillips got um (0.5) knee:
2 wa:     lking dru::nk at the reception.
3       → (514 ms)
4 Mom:    Who:?
5 Bet:    Phillips,

The density plots in this section present the durations of the transitions between trouble-source TCUs and OIRs along the *x*-axis. Positive values constitute gaps and negative values are overlaps. The density curves represents estimates of the frequency of cases with a given transition time. The peak of the curve corresponds to an estimate of the mode of the distribution.

A comparison between the timing of OIRs and responses to polar questions is presented first (see OIRs and Responses to Polar Questions), after which two comparisons within OIR types are presented: open versus specific (see Open and Specific OIRs) and corrections versus non-corrections (see Corrections and Non-Corrections).

#### OIRs and Responses to Polar Questions

**FIGURE 1:**
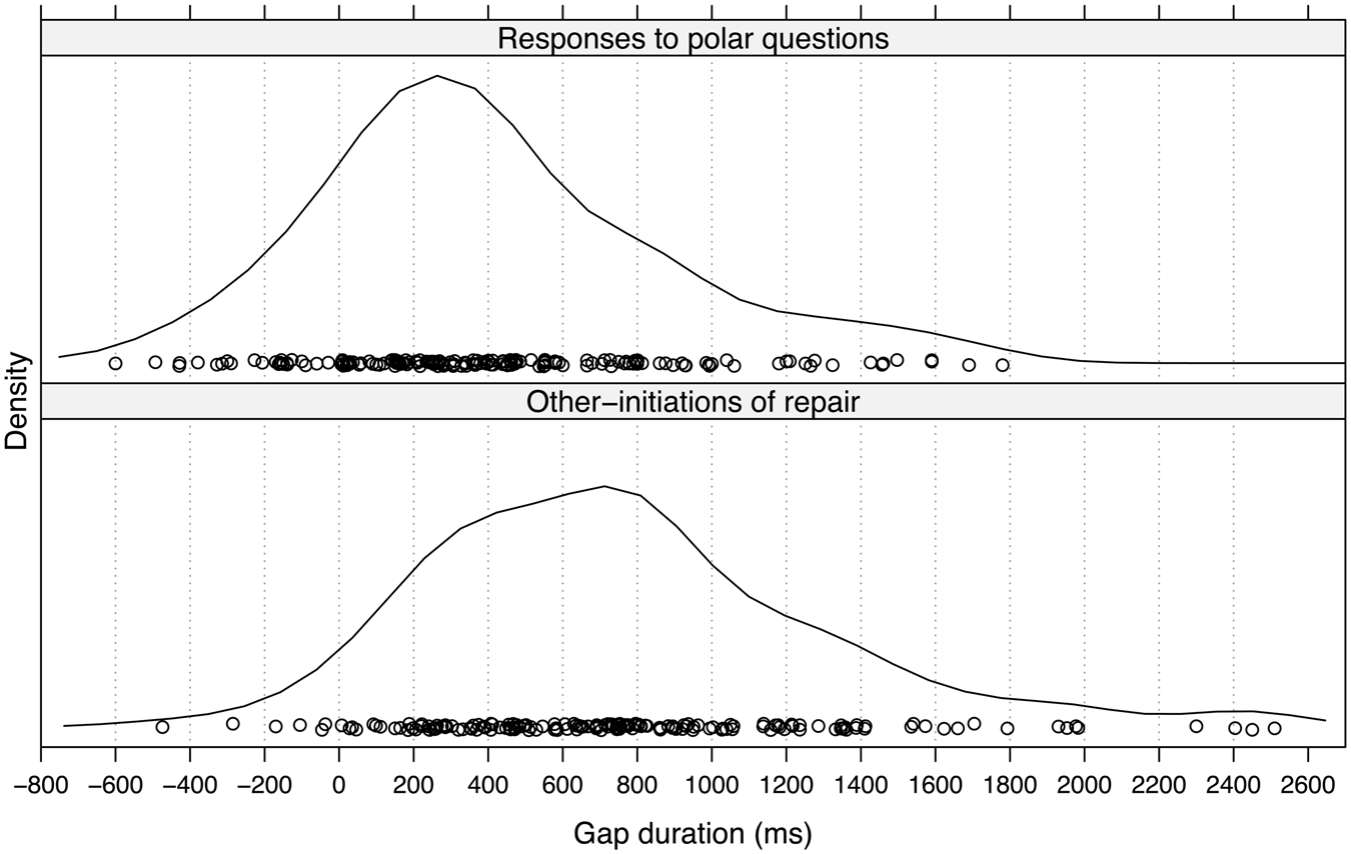
**Gap durations (in milliseconds) for other-initiations of repair (OIRs) and responses to polar questions**.

**Figure 1** presents a density plot of the gap durations for OIRs (*n*= 169) and responses to polar questions (PRs; *n*= 169). An inspection of the two distributions reveals that OIRs tend to occur after significantly longer gaps that PRs. The mode gap duration for OIRs is ∼700 ms, whereas the mode for PRs is roughly 300 ms. A Wilcoxon Mann–Whitney Rank Sum Test confirms that the distribution of OIRs tends to have larger values than that of PRs (*Z* = 6.5228, *p* < 0.001, *r* = 0.5). Additional descriptive statistics are given in **Table 2**.

**Table 2:** Descriptive statistics (mean, standard deviation, and median in milliseconds) for gap durations by type.

	Mean (SD)	Median	*N*
Responses to polar questions	397 (475)	339	169
All OIRs	760 (532)	721	169
Specific OIRs	726 (568)	633	116
Open OIRs	835 (439)	787	53
Corrections	412 (422)	274	12
Non-corrections	787 (530)	744	157

The analysis of the timing of OIRs in general supports the conclusion that OIRs systematically occur after long gaps. The most frequent OIRs do not occur within the same timing window the most frequent PRs, between 0 and 500 ms, but few OIRs occur after 1500 ms. Indeed, if one assumes that the timing of PRs serves as a good proxy for a normal turn transition, as has been argued (Stivers et al., 2009), then the analysis suggests that in this data OIRs typically occur after 400–500 ms of delay beyond the 300 ms duration of a normal transition space.

#### Open and Specific OIRs

**FIGURE 2:**
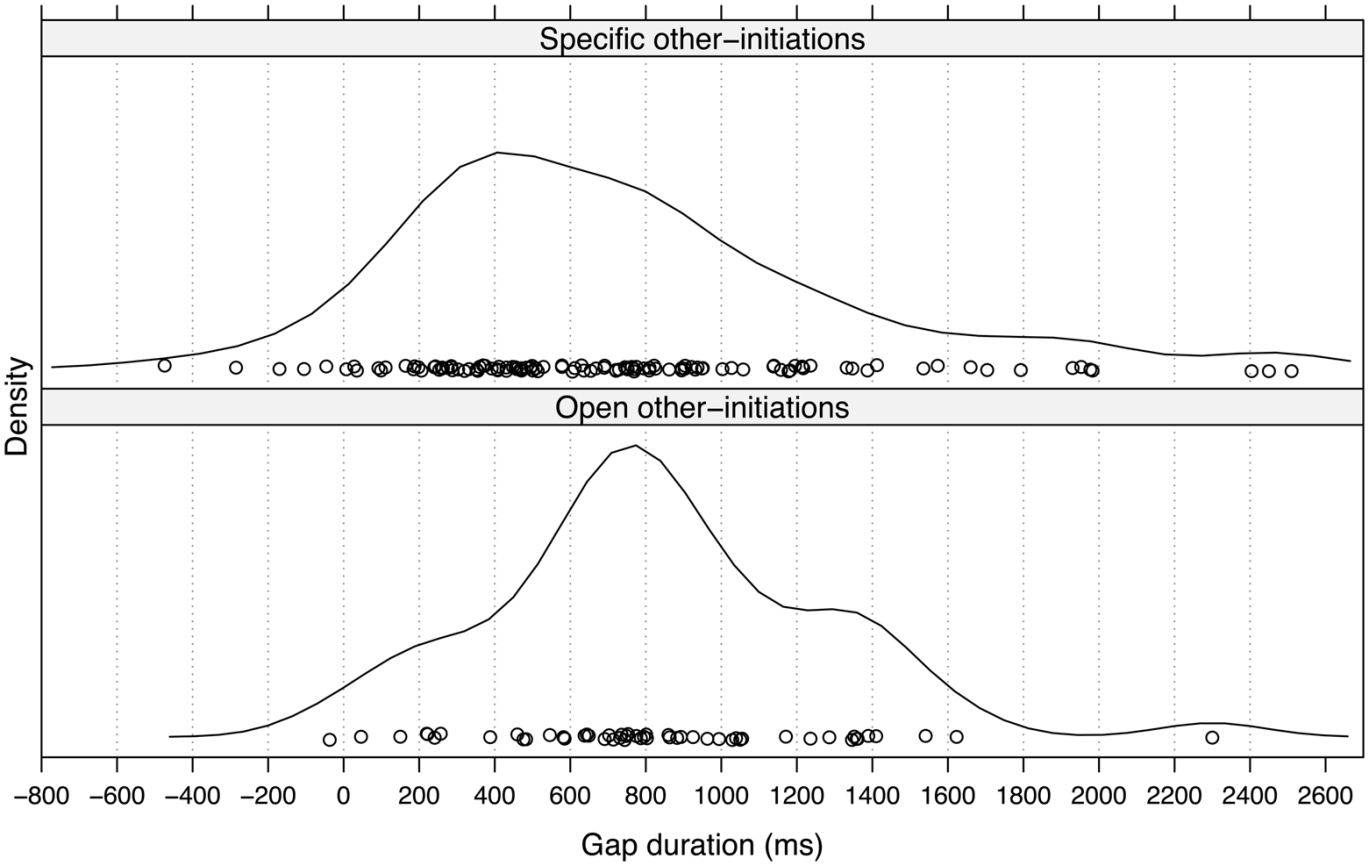
**Gap durations (in milliseconds) for open and specific OIRs**.

The density plot in **Figure 2** shows the distributions of gap durations for open (*n =*53) and specific OIRs (*n*= 116) in next position. The density curves indicate that the most frequent gap duration for open OIRs is between 700 and 800 ms, in contrast to approximately 400 ms for specific OIRs. A Wilcoxon Mann–Whitney Rank Sum Test indicates that the two distributions differ significantly (*Z* = 1.97, *p* < 0.05, *r* = 0.15). These results suggest that on average speakers of open OIRs delay ∼300–400 ms more than speakers of specific OIRs.

#### Corrections and Non-Corrections

**FIGURE 3:**
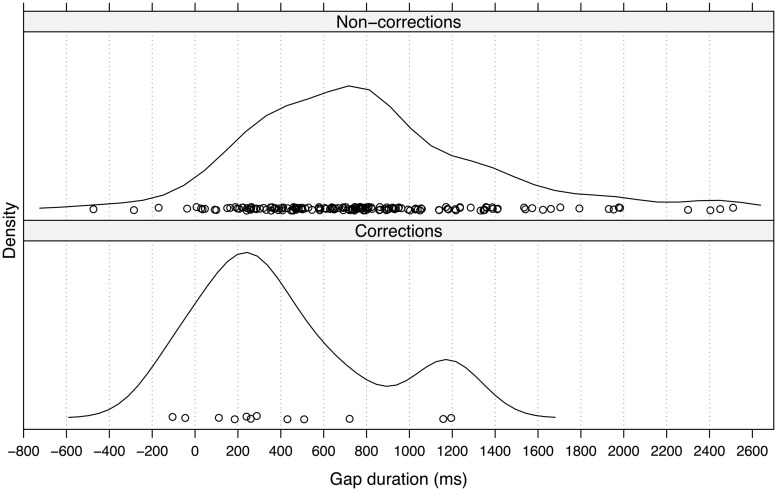
**Gap durations (in milliseconds) for corrections and all other OIRs (non-corrections)**.

The frequency of other-corrections in the corpus was low, with only 14 cases in total and only 12 cases in next position to the trouble-source TCU (see Materials and Methods). **Figure 3** presents a density plot for the gap durations of other-corrections (*n*= 12) and all other OIRs (*n*= 157) in next position. An inspection of the density plot reveals that, although the number of cases in the collection is small, other-corrections tend to occur earlier than OIRs in general. The most frequent gap duration for other-corrections is between 200 and 300 ms whereas other OIRs most frequently occur after ∼700–800 ms. A Wilcoxon Mann–Whitney Rank Sum Test indicates that other-corrections tend to have shorter gap durations (*Z* = -2.64, *p* < 0.01, *r*= 0.20). These results suggest that in contrast to the bulk of other-initiations, other-corrections do not occur after significant delay and in fact occur within a similar temporal window as responses to polar questions.

### What do Speakers Use the Transition Spaces Before OIRs to Accomplish?

The analysis of the timing of OIRs revealed that the most frequent cases occur after gaps of ∼700 ms, in contrast to 300 ms for responses to polar questions. This observation raises the question of what participants use this extra time to accomplish. A qualitative analysis of the transition spaces before OIRs points to three possible answers. The speaker of an OIR, before its production, can:

(1) perform a search for late recognition of the trouble-source turn,
(2) provide an opportunity for the speaker of the trouble-source turn to self-initiate repair, and
(3) produce visual signals, such as facial gestures, that display a lack of recognition and thereby occasion—if not initiate—self-repair.

As the analyses in this section make clear, these possibilities are not necessarily mutually exclusive.

#### Searching for Late Recognition

In order to produce a relevant next turn, a next speaker must hear, and understand the current turn. The fact that next turns frequently take only 100–300 ms to initiate suggests that the procedure next speakers engage in to recognize the current turn’s meaning and action typically occurs quickly, enabling a minimization of gaps between turns (Levinson, 2013). But recognition does not always occur so quickly and can in fact come late, even after a next speaker displays a lack of recognition. This can be seen in the following cases, in which the recognition of a prior turn occurs *after* the next speaker initiates repair.

(6) RCE06
1 Alex:    Did you like buy some lemonade earlier,
2          (937 ms)
3 Rob: →  Buy some what,=<lemonade,
4 Alex:    (Yeah)
5 Rob:     Yeah yeah yeah.
(7) Virginia
1 Wes:    (Now) you taught ’er howda dance, didn’ you?
2         (1236 ms) 
3 Vir: →  Huh? [ (.) [Yeah.
4 Wes:         [Weren[’t you teachin’ er’ some new
5         steps the other day?
6 Vir:    Y:eah.

In the first case, Rob apparently fails to hear a word in Alex’s question, evidenced by his OIR (“buy some what,”), which locates “lemonade” as a trouble source and makes repetition of this word by Alex conditionally relevant. But before Alex responds, Rob initiates self-repair, producing the very word he claimed, by virtue of his OIR, not to have heard. The word is produced with a prosodic practice known as a left push, noted by the “<” in the transcript, through which the speaker interdicts the relevance of transition at the completion of a prior TCU (cf. Local and Walker, 2004). Here the left-push is hearable as a ‘last millisecond’ effort to get the word into the turn before the other responds. In the second case, Virginia apparently fails to hear Wes’s question and initiates repair with “Huh?.” Then, a moment later, after a micro pause of 140 ms, she answers the question in overlap with Wes’s self-repair, claiming in effect to have heard the question, at least well enough to confirm it. These cases suggest that in addition to a procedure that results in ‘immediate’ recognition of a current turn, next speakers also have available a procedure that can result in ‘late’ recognition.

Although a search for late recognition is primarily a private process, observable behaviors, such a momentary withdrawal of gaze in the transition space, may reflect this process and thereby render it public. Psycholinguistic research has shown that speakers often avert their gaze when asked questions and that this in turn facilitates remembering and speech planning (Glenberg et al., 1998; Doherty-Sneddon and Phelps, 2005; Markson and Paterson, 2009). Thus the withdrawal of gaze in the transition space before a relevant next turn may be a public exponent of a private search for recognition, as is arguably the case in the next extract. Here, after Heather initiates a new sequence, assessing a taxi driver that she evidently hired the night before, Kelly looks away from Heather in an expanded transition space, apparently engaged in a search for recognition.

**FIGURE 4:**
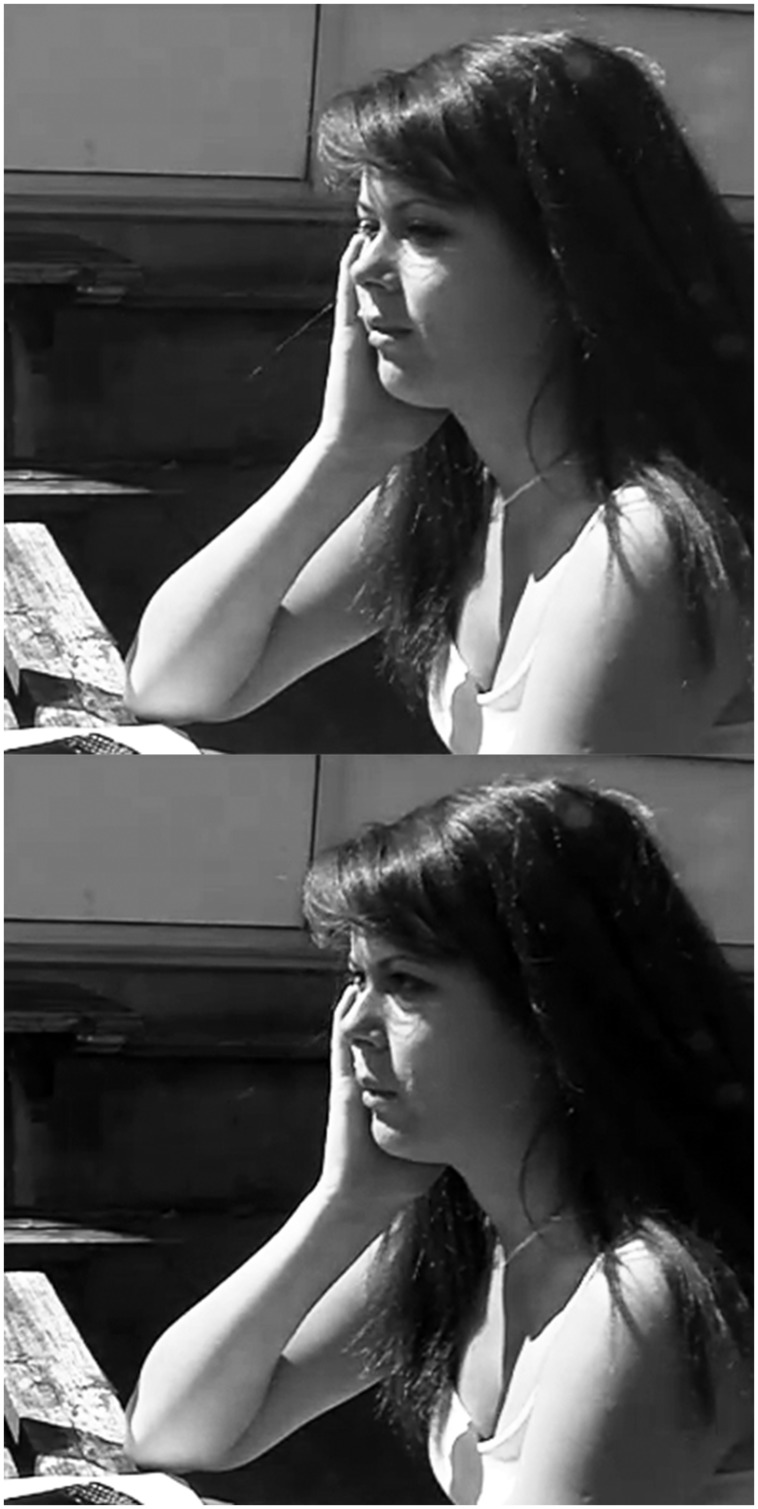
**Kelly looks away at the completion of the trouble-source turn and holds this position for ∼1100 ms **(top)**.** She then returns her gaze to Heather for ∼250 ms before she produces the OIR **(bottom)**.

(8) RCE28
1 Hea:    That taxi driver last night was really
2         friendly.
3    →   (1346 ms) ((see **Figure 4**))
4 Kel:    What?
5         (0.6)
6 Hea:    My taxi driver was really friendly.
7         (0.4)
8 Kel:    OH, yea[h.
9 Hea:           [yesterday.
10        (1.5)
11 Kel:   I was like she took a while.

The reference to the taxi driver in Heather’s turn includes a demonstrative (“that taxi driver”), signaling to Kelly that she should be able to recognize the reference (Himmelmann, 1996). At the completion of Heather’s turn, Kelly averts her gaze from Heather and holds this position for ∼1100 ms (see **Figure 4**). The timing of Kelly’s look away coincides with the recognizable completion of Heather’s turn and thereby shows that Kelly has heard the turn, at least well enough to identify a transition relevance place (Sacks et al., 1974; cf. Holler and Kendrick, 2015). The look away also shows that Kelly has begun to act at just the place where an action by her is relevant. In this way, the withdrawal of gaze at a transition relevance place can be seen as preparatory to an incipient response. In this case, however, Kelly does not produce a relevant response, but rather returns her gaze to Heather and initiates repair. One plausible account of this behavior is that the withdrawal of gaze reflects a search for recognition, one that evidently fails. A search for late recognition is thus one possible use that a next speaker can make of an expanded transition space, one which, if successful, may obviate the need for repair.

#### Providing an Opportunity for Self-Initiated Repair

An expansion of the transition space, whether the result of a search for recognition or not, has as an interactional affordance the provision of an opportunity for the current speaker to self-initiate repair and thereby potentially resolve the trouble (Schegloff et al., 1977). Indeed, the absence of a response within a normal transition space can occasion a self-initiation of repair by the current speaker, as can be seen in the following extract.

(9) Monopoly Boys
1 Rick:    You have th:e thing I need for the mono:poly.
2          (540 ms)
3 Rick: →   Over there. ((points))
4          (438 ms)
5 Rick: →   The reds.
6 Luke:     Oh, yeah.

In the course of a game of Monopoly, Rick notices that Luke has “th:e thing” (i.e., a specific property) that he needs in order to have a monopoly in the game (i.e., to own all the properties of a specific color). At the word “thing” Luke can be seen to begin a visual search of his properties (a set of cards on the side of the game board) that continues into the transition space after Rick’s turn. Rather than wait for Luke either to resolve the reference himself or to initiate repair (e.g., with “what” or “what thing”), Rich adds an increment to his turn, which, together with a deictic pointing gesture, specifies the area in Luke’s visual field he should search and thereby assists him in the resolution of the problematic reference. After this, too, fails to secure recognition from Luke, Rick again initiates self-repair, replacing “th:e thing” with “the reds” (i.e., the red colored properties), a form of indexical repair that speakers can employ to pursue a response (Bolden et al., 2012). With this, Luke is apparently able to resolve the problematic reference and registers this change of state publicly with “oh” (Heritage, 1984) and confirms Rick’s noticing with “yeah.”

Here, then, the self-initiation of repair by the current speaker is an alternative to the other-initiation of repair by next speaker. Luke uses the transition spaces that emerge in the course of Rick’s turn to search for recognition, a search that, in this case, is publicly observable. Before the search comes to an end, either in late recognition or failure (i.e., an other-initiation of repair), Rick uses the transition spaces as opportunities to self-initiate repair. The practices that current and next speakers employ reveal complementary orientations to their accountability for the intelligibility of the current turn (Garfinkel, 1967). Luke does not initiate repair immediately; he withholds other-initiation to first search for recognition independently. In this way, he holds himself accountable for the recognition of the current turn. Likewise, Rick does not wait for Luke to initiate a repair procedure; he self-initiates repair at the first sign that the recipient has failed to recognize his turn (i.e., the expansion of the transition space and the visible search). In so doing, he orients to his accountability for the intelligibility of his own conduct.

If the current speaker passes on the opportunity to self-initiate repair provided by an expansion of the transition space, the necessity to find a resolution of the trouble falls to the next speaker. That is, if the current speaker does not initiate self-repair, the next speaker may resort to OIR, as Rich does in the next extract.

**FIGURE 5:**
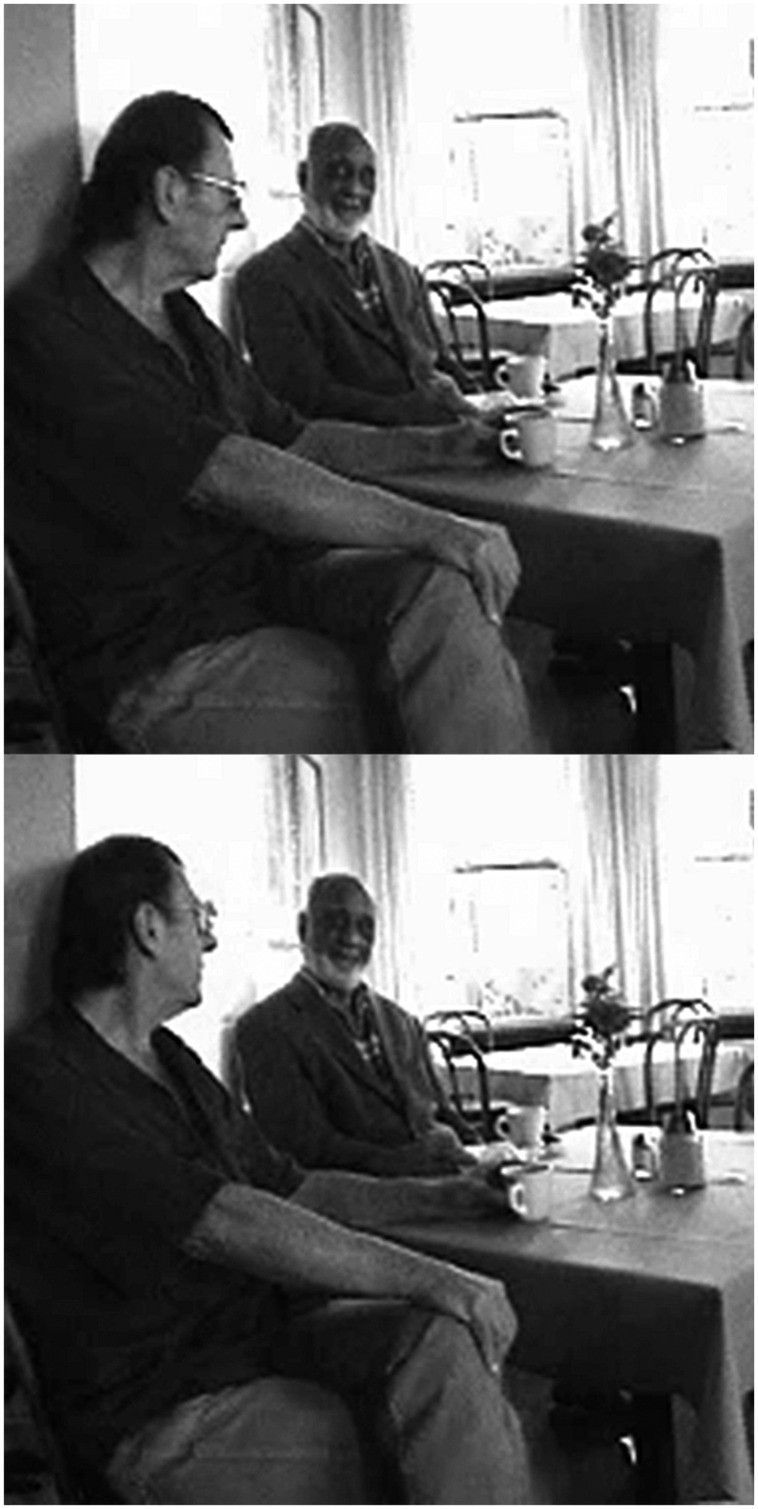
**Rich, on the left, looks down during the trouble-source turn **(top)**.** He then turns to look at Tom after the trouble-source turn is complete and holds his gaze on Tom for ∼800 ms before he produces an OIR **(bottom)**.

(10) Coffee Chat (simplified)
1 Rich:    ((clears throat))
2          (2.0)
3 Rich:    WE[:LL,
4 Tom:     [That’s in: building A?
5(1286)    ((see **Figure 5**))
6 Rich: →  Pardon?
7 Tom:     What building are you in?
8 Rich:    Yeah: I’m on the second floor A building.

After a lapse in the conversation, in which Rich can be seen to inspect his empty coffee cup, a possible warrant to leave the table, Rich produces what can be heard as a preliminary to his departure from the interaction (“WE:LL,” at line 4). In overlap with this, Tom poses a question to Rich that continues on the topic of the talk from before the lapse (i.e., who lives in the same building as Rich in a retirement community). During the question, Rich looks down and forward. ∼300 ms after Tom’s question comes to completion, Rich turns his head to the left to direct his gaze at Tom (see **Figure 5**). He maintains this position, gazing at Tom, for roughly 800 ms before he produces an OIR (“Pardon?”, line 7).

While the entire duration of the transition space before the OIR constitutes an opportunity in which Tom *could* self-initiate repair (e.g., by repeating his question), the roughly 800 ms that Rich holds his gaze on Tom arguably constitutes a space in which Tom *should* self-initiate repair. Heath (1984, p. 253) has argued that gaze, as a display of recipiency, is “sequentially implicative” and “declares an interest in having some particular action occur in immediate juxtaposition with the display.” In line with this, Rossano (2006) and Stivers and Rossano (2010) have argued that participants use gaze to signal the relevance of a response. The withdrawal of gaze, in contrast, has been associated with an absence of sequential implicativeness, both at possible sequence completion (Rossano, 2012) and in word searches (Goodwin and Goodwin, 1986). Thus although the current speaker has an opportunity to self-initiate repair and although the deployment of gaze by next speaker arguably displays an expectation that the current speaker should act, the current speaker here passes on the opportunity to self-initiate repair. The initiation of the repair procedure then falls to the next speaker.

#### Producing Visual Signals

An expansion of the transition space before the production of an OIR also provides space for, and can be the result of, a next speaker’s production of visual signals and other visible bodily actions. It has been shown that head movements, such as a lateral tilt or forward extension of the head, can serve to occasion self-repair (Seo and Koshik, 2010) and that particular body movements frequently occur in repair sequences (Rasmussen, 2013; Li, 2014; Floyd et al., in press). In addition, facial gestures, like raising or furrowing one’s eyebrows, can be preliminaries to verbal OIRs. In the following extract, after Abbie turns to gaze at Maureen, she raises her eyebrows and holds this position for ∼260 ms before she initiates repair with “Hm:?”

**FIGURE 6:**
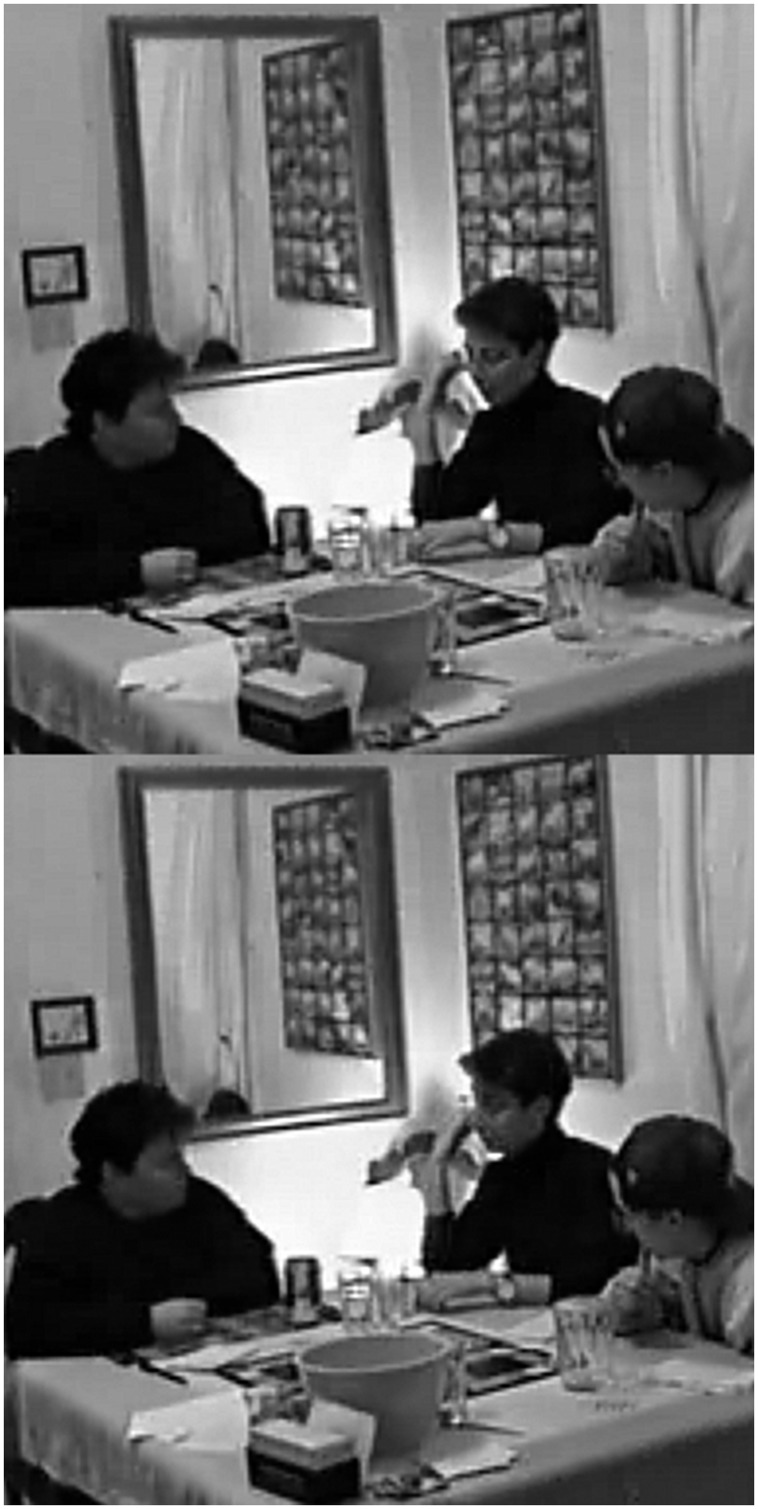
**Abbie, in the middle, looks down during the trouble-source turn **(top)** and then raises her eyebrows and turns to look at Maureen.** She holds this position for a beat (∼260 ms) before she produces an OIR **(bottom)**.

(11) Game Night
1 Abbie:    Apparently she’s a really spiritual person
2           with a lot of spirituality and stuff like
3           ↑this..hh
4 Maureen:  M.A.?
5           (714) ((see **Figure 6**))
6 Abbie: →  Hm:?
7 Terry:    Mm:hm[:,
8 Maureen:  [Is it M.A.?
9 Abbie:    Mm:.

The trouble source that Abbie’s OIR locates is itself an OIR which locates the reference to “she” at line 1 as a trouble source and offers the initials “M.A.” as a candidate replacement. Abbie’s conduct in the transition space – directing her gaze to Maureen, raising her eyebrows, and holding for a beat – not only provides an opportunity for the self-initiation of repair but also constitutes a visible and accountable signal, in the form of a facial gesture, that displays a lack of recognition and a state of recipiency. (Note that Abbie’s open OIR “hm:?” lacks the “astonished” prosody associated with open OIRs that signal surprise; Selting, 1988.) In this case, the visual signal does not itself elicit a self-repair; the next speaker goes on to produce a verbal OIR. But elsewhere such visual signals can prompt a current speaker to self-repair his or her talk without a verbal OIR.

In the next extract, Heather self-repairs a place reference in her answer to Kelly’s question after Kelly produces a facial gesture that displays a lack of recognition. The question that Kelly asks Heather concerns the amount of time that a friend of Heather’s has lived in specific regions of England.

**FIGURE 7:**
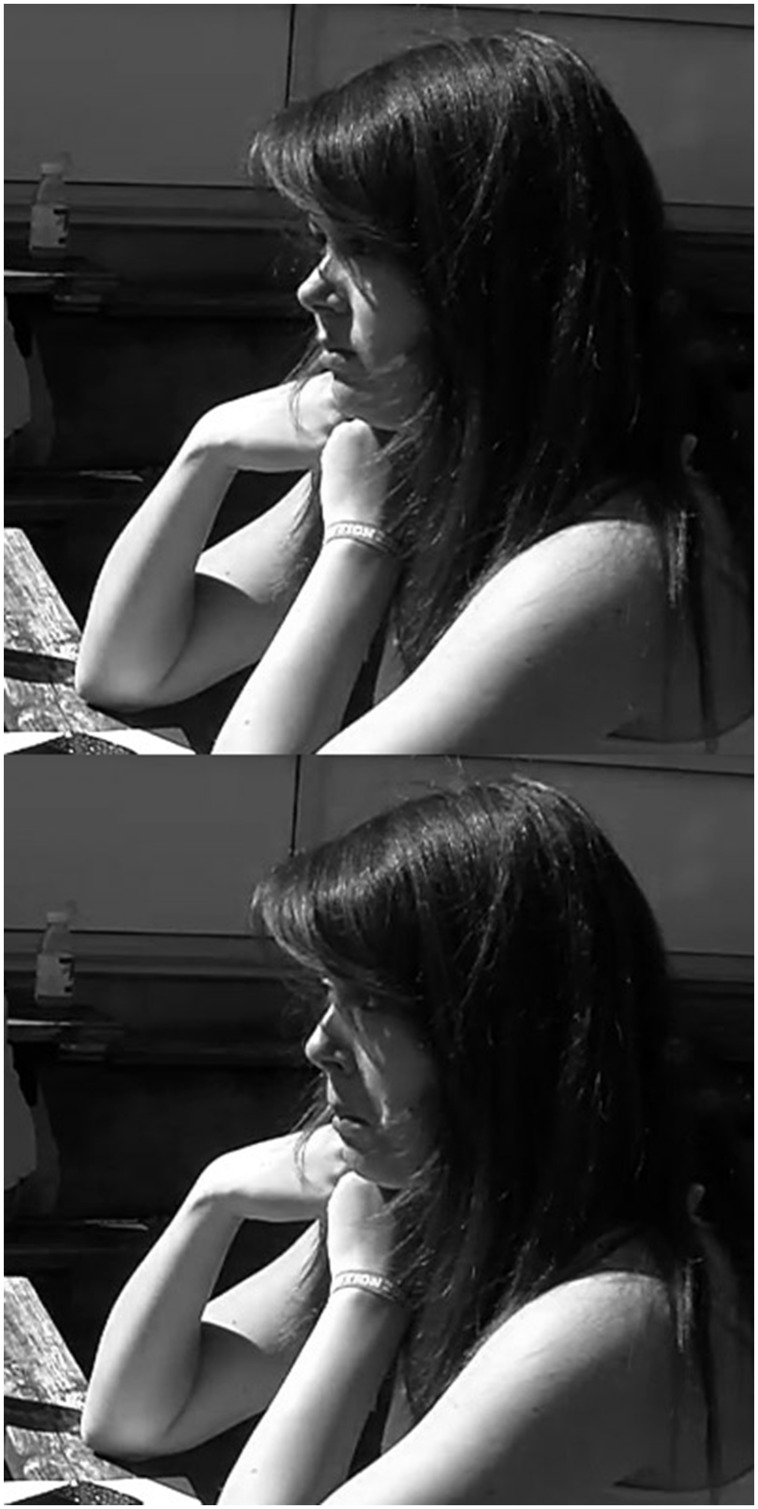
**After Heather’s answer to Kelly’s question is complete, Kelly gazes at Heather and holds this position for ∼840 ms **(top)**.** She then raises her eyebrows, pulls down the corners of her mouth, and holds this facial gesture for ∼510 ms **(bottom)**, until Heather produces a self-repair.

(12) RCE28
1 Kel:    It’s over ten years, that’s pretty much all::
2 Hea:    Yeah he went to my infant school and he went
3         to:: St. Jo:se:ph which is like the school in
4         hhh (0.8) well I think it- (.) counts as
5         Merrow.
6    →   (1350 ms) ((see **Figure 7**))
7 Hea:    or like Guildford.=But it’s [still Surrey.
8         (0.2)                       [((Kelly nods))
9 Kel:    (Okay)

After some initial trouble with the place reference, Heather offers “Merrow” as the name of a place where the friend has lived. In a position in which acceptance of the answer is relevant (e.g., a sequence-closing third, see Schegloff, 2007), a gap of ∼840 ms emerges, at which point Kelly produces a facial gesture – raising her eyebrows and pulling down the corners of her mouth – displaying a lack of recognition (see **Figure 7**). Kelly holds this facial gesture for ∼510 ms, until Heather self-repairs the place reference from “Merrow” to “Guildford,” a nearby town. The reference to “Guildford” is apparently recognizable to Kelly, who begins to nod shortly thereafter, and after Heather offers “Surrey” as the name of the county where both places reside, Kelly accepts the answer as adequate and brings the sequence to a close (line 9). This case demonstrates that a facial gesture by a possible next speaker can be sufficient to occasion self-repair. The production of a visual signal within the transition space before an OIR, such as in Extract (11), could therefore be a practice for the resolution of a trouble, one that may obviate the need for a verbal OIR^1^.

## Discussion

### The Motivations for and Consequences of Delay Before Other-Initiations of Repair

The quantitative analysis of the timing of OIRs in conversation confirms the observation that OIRs tend to occur after expanded transition spaces. Indeed, if one assumes that the timing of responses to polar questions can serve as a proxy for a normal turn transition, as others have done (Stivers et al., 2009), then the results suggest that other-initiations typically occur after 400–500 ms of delay beyond the 300 ms duration of a normal transition space. But why should this be so? The explanation put forward by Schegloff et al. (1977), discussed previously, is that next speakers who encounter troubles of speaking, hearing, or understanding regularly *withhold* OIR to provide an opportunity for self-initiations of repair. The nature of this explanation is unclear, however. Is this an explanation of a personal motivation for the delay? That is, does a next speaker withhold an OIR *in order to* create an opportunity for the current speaker to resolve the trouble? Or is this an explanation of a public consequence of the delay, one that leaves the question of motivation unanswered? In principle, a delay before an OIR is an opportunity for a self-initiation of repair, whatever its cause.

The analysis of what speakers use the transition spaces before OIRs to accomplish suggests that providing an opportunity for self-initiation of repair does not exhaust the set of possible motives for delay. Although in some cases one can argue that such a motive may lie behind the observed delay (see Providing an Opportunity for Self-Initiated Repair), others point to alternative explanations. The fact that next speakers who initiate repair do, on occasion, recover all, or part of the trouble source after an OIR demonstrates that ‘late’ recognition is possible. Together with observations of subtle visible bodily actions, such as gaze aversion in an expanded transition space, the qualitative evidence suggests that next speakers who fail to hear or understand the trouble-source turn can engage in a search for this late recognition (see Searching for Late Recognition). The motivation for the delay in such cases is not to provide an opportunity for self-initiation of repair, but more proximally to resolve the trouble independently, without recourse to an OIR that exposes the trouble and stops the progressivity of the sequence. The evidence also suggests that the visual signals such as facial gestures can precede verbal OIRs. These and other visible bodily actions, including reorientations of the head and torso, take time to produce and can cause a delay – measured in milliseconds of silence – before an OIR. Here, too, the motivation for the delay does not directly concern the self-initiation of repair.

But regardless of the motivation, the consequence is the same: an expanded transition space before an OIR can be a covert signal of trouble and can provide an opportunity for the current speaker to self-initiate repair. Although one can interpret Schegloff et al.’ (1977) explanation as an account of a personal motive, their commitment to uncovering abstract properties and principles of interactional systems suggests that their target was not the individual and his or her motives, but rather an orderliness that transcends such personal concerns. While the results of the investigation are compatible with their explanation, research on the timing of the self-initiation of repair, in particular so-called transition space repairs, is necessary to confirm it. The model that Schegloff et al. (1977). propose predicts that transition space repairs should occur earlier in the transition space than other-initiations. If so, it would provide evidence for a system in which the temporal window for self-initiation precedes that for other-initiation.

Although the results of the investigation are of primary relevance to models of turn-taking and repair in conversation analysis, they may also be of interest to psycholinguists, for whom the timing of turn-taking presents a puzzle. Given that experimental research has shown that speakers need at least 600 ms to plan even a simple word (Indefrey and Levelt, 2004; Indefrey, 2011), processes of language production in conversation must begin well before the current turn ends (Levinson, 2013). Psycholinguists have thus begun to investigate the cognitive processes that enable the minimization of gaps between turns (Magyari and de Ruiter, 2012; Magyari et al., 2014; Sjerps and Meyer, 2015). The results of the current investigation, however, remind us that estimates of central tendency hide a wealth of complex variation, as a number of factors, such as the type of turns involved, influence the timing of transitions. To psycholinguists, the 700 ms of silence that precedes OIRs might be taken to reflect a cognitive process – comprehension gone awry. Indeed, the search for late recognition, as I have called it, may be just such a process. But in face-to-face conversation, the core ecological niche of language (Schegloff, 2006), the line between cognitive processes and socio-interactional ones is blurred. Visible bodily actions, such as an aversion of gaze or a facial gesture, can render otherwise private processes public, at which point they may feed into socio-interactional ones. Even timing alone – a recognition that a speaker has not produced a turn when it was due – can occasion actions such as self-repair. In this way, the private and the public are woven together in an interactional system, and it is within such a system that the silence that precedes OIRs must be understood.

### The Preferences for Self-Correction and Specificity

The properties and principles of the repair system, Schegloff et al. (1977) argued, maximize opportunities of self-initiated repair, which come early, and often, and minimize opportunities for other-initiated repair, which as we have seen tend to come late in the transition space. This institutionalized bias in the repair system is known as the preference for self-correction. As evidence of this, Schegloff et al. (1977, p. 379). claimed that other-corrections exhibit special marking and special positioning (e.g., the qualification of epistemic stance or delay within a turn or sequence) that orient to a dispreferred status. With respect to the position of other-corrections within a turn, however, the current investigation finds no evidence for an orientation to dispreference. The results of the analysis, while based on a small sample, show that the other-corrections in the corpus tend to occur without delay, most frequently after 200–300 ms. This suggests that speakers do not withhold other-corrections to provide an opportunity for self-correction. Moreover, the claim that other-corrections typically include qualifications or modulations of epistemic stance has also recently been called into question (Haakana and Kurhila, 2009). Taken together, these findings cast doubt on the status of other-correction as a dispreferred action and suggest that further investigation, based on a larger sample of cases, is warranted.

The relevance of these results to the preference for self-correction itself is less clear. Other-corrections are relatively rare. The entire corpus contains 222 other-initiations, including those that occur after next position; only 6% (*n*= 14) of these are other-corrections. This suggests that many opportunities that speakers may have had to issue a correction simply were not taken. Moreover, other-corrections appear to be restricted to specific types of trouble sources. Of the 14 cases of other-correction, nine locate proper names, or numbers as trouble sources and three target mispronunciations or malapropisms (e.g., “antioxidities” rather than antioxidants). In contrast, other practices for other-initiation do not appear to be restricted in this way. Thus although other-corrections may not be constructed as dispreferred actions (i.e., with delay or qualification), a restriction of other-corrections to specific contexts may nonetheless be evidence of a systematic bias against their use.

In addition to the preference for self-correction, Schegloff et al. (1977) also argue for a preference for specificity in the selection of OIR practices, such that more specific (or ‘stronger’) other-initiations are preferred over less specific (or ‘weaker’) ones (cf. Clark and Schaefer, 1987). Two pieces of evidence are given to support this claim. First, if an other-initiation is subject to self-repair within the same turn, the self-repair occurs from a less to a more specific format, but not the inverse. Second, if more than one other-initiation is needed to resolve the trouble, speakers use increasingly specific practices. The current investigation adds two additional pieces of evidence for a preference for specificity. Third, specific other-initiations are more frequent than open other-initiations (only 31% are open, see **Table 2**; cf. Kendrick, in press). And fourth, specific other-initiations tend to occur earlier in the transition space than open other-initiations, in line with the tendency for dispreferred alternatives to be delayed.

The observation that some types of OIRs occur after less delay than others also opens up new avenues for future research. Within the diversity of specific OIRs, for example, one may discover systematic variation. The precise timing of an OIR could indicate a particular epistemic stance, such as whether the OIR signals a trouble of hearing or understanding *per se*, or whether it displays a speaker’s surprise or disbelief (see Identification of OIR).

### The Intersection of Turn-Taking and Repair

The model of turn-taking that Sacks et al. (1974) proposed accounts for the minimization of gaps in conversation through a set of rules and constraints that motivate fast transitions between turns. Given that OIRs are themselves turns at talk, the observation that OIRs tend to occur after relatively long gaps would therefore appear to undermine this model. In this section – an exploration of the intersection of turn-taking and repair – I first outline a series of systemic constraints on the timing of next turns and then argue that OIRs supersede them, an argument first made by Sacks et al. (1974) and Schegloff et al. (1977), but only partially articulated in their work.

To begin, consider the initial boundary of the transition space. The turn-taking system includes a constraint against more than one speaker at a time, and while more than one speaker at a time is common, it is an unstable state, one which quickly resolves back to a single speaker (Schegloff, 2000). This accounts for the observation that next turns tend to start up at or near possible completions of prior turns, where transition can occur without (or with minimal) violation of the constraint, not sooner.

At the final boundary of the transition space, there are at least three constraints in operation, two of which are rooted in the rules for turn allocation, which provide a motivation for fast transitions (Sacks et al., 1974). Roughly, if no one has been selected to speak next (e.g., by an addressed question), a speaker may self-select to take a turn. If more than one speaker self-selects, the first to start has rights to the turn and the second starter should cede the turn to the first. These rules establish a motivation for next speakers to start up early and thereby minimizes the gaps between turns.

If no one self-selects, however, the current speaker may continue his or her turn. The possibility that such a continuation may be imminent also provides for the minimization of gaps, as next speakers aim to begin before this occurs. Although the time course of this rule is unknown, a computational corpus study of Dutch telephone conversations by Bosch et al. (2005) provides a useful estimate. The duration of silences between utterances within a turn was found to be greater than the duration of silences between turns, with mean durations of 520 and 380 ms, respectively. This is compatible with a model of turn-taking in which an opportunity for self-selection by next speaker temporally precedes that for continuation by current speaker. The expanded transition spaces before OIRs are therefore the result of a connivance: the next speaker passes the opportunity (or obligation, in the case of current-selects-next) to speak, and the current speaker, the one who produced the trouble source, passes the opportunity to continue the turn. At ∼700 ms, the average other-initiation of repair occurs after the absence of a continuation by the current speaker would be recognizable. Indeed, there is evidence that 700 ms may in fact be a generic threshold in conversation (Kendrick and Torreira, 2012, 2014; Roberts and Francis, 2013), perhaps for this very reason.

A third constraint at the final boundary of the transition space is grounded not in the rules for turn allocation but in the potential for silences in conversation to become meaningful, as signals of interactional trouble (Jefferson, 1986, 1983a; Pomerantz, 1984; Schegloff, 1988; inter alia). In an adjacency-pair sequence, to cite but one context, even a slight delay beyond a normal transition increases the likelihood that the second pair part will have a dispreferred turn format, and a long delay, on the order of 700–800 ms, is a reliable signal that a dispreferred response is imminent (Kendrick and Torreira, 2014). The semiotics of silence is therefore an additional basis for a constraint on the timing of next turns, one that, like the rules for turn allocation, creates a bias toward fast transitions and the attendant minimization of gaps.

Given the existence of systemic constrains on the timing of next turns and the observation that OIR occur after significantly longer gaps than other turns, one solution to this apparent puzzle naturally presents itself: OIRs may trump the rules of the turn-taking system. Indeed, this appears to be the tack taken, though only partially articulated, by Sacks et al. (1974) and Schegloff et al. (1977). The timing of OIR, they argued, reveals “the independent status of the repair organization, whose operation may supersede otherwise operative aspects of the turn-taking organization” (Schegloff et al., 1977, p. 374). Although they do not elaborate this point, they do provide one additional example. In a discussion of second-starter supersession (i.e., methods whereby a second speaker to self-select may win the turn), Sacks et al. (1974, p. 720) observe that “when a self-selector’s turn-beginning reveals his turn’s talk to be prospectively addressed to a problem of understanding [a] prior utterance, he may by virtue of that get the turn, even though at the turn-transfer another started before him.” In other words, a second speaker to self-select takes priority if he or she produces an other-initiation of repair. This, then, is evidence that participants in conversation orient to resolving troubles of hearing and understanding as a “priority activity” (Sacks et al., 1974, p. 720), one which takes precedence over rules of the turn-taking system that motivate fast turn transitions.

A consequence of the priority of OIR, one which to my knowledge has not previously been registered, is that OIRs may freely start up in overlap with a post-trouble-source turn or TCU, and need not employ the practices for overlap competition described by Schegloff (2000), such as increases in volume, speech rate, or pitch. In each of the cases below, an OIR starts up in overlap with a turn or TCU that intervenes between it and the trouble-source TCU. Although the OIR is not designed as competitive, in each case the speaker of the prior turn or TCU drops out, ceding the turn to the speaker of the OIR.

(13) KC-4:2 (Sacks et al., 1974, p. 720)
1 R:       Hey::, the place looks different.
2 F:       Yea::hh.
3 K: →     Ya have to see ou[r new-
4 D: →                      [It does?
5 R:       Oh yeah
(14) WG 4-13-nh (Benjamin, 2013, p. 188)
1 Hal:     he may be victimized on it
2          (0.6)
3 Hal: →   I’m not sure he’s ma[king]
4 Nix: →                       [you ] mean by his lawyer
5 Nix:     [hhhhhhhhh]
6 Hal:     [yeah or] (.) somebody else
(15) CallHome 6079 (Benjamin, 2013, p. 119)
1 A:      it was [so: nice] it was so nice =
2 B:      [hhhhh @ ]
3 A:      = that they came I can’t even tell you
4         like.hhhh like (0.6) just seeing them
5         like I was performing to them
6         (.)
7    →    like I was sm[iling at th]em like
8 B: →                 [to who. ]
9 A:      .hhhh Juliette, Sam: and (.) and Tara

The fact that OIRs win the turn without the need for competition and, moreover, that trouble-source speakers respond to OIRs with no delay is further evidence that repair is a priority activity in conversation (see also Extract 4).

The data above also illustrate yet another intersection between turn-taking and repair. As noted previously, the imminent possibility that a current speaker may continue his or her turn if no one self-selects builds a motivation for fast transitions into the turn-taking system. Note, however, that the OIRs in Extracts (14) and (15) occur in overlap with a continuation by the current speaker (at the first arrowed lines). This demonstrates that OIRs supersede not only first-starters in self-selection, but also continuations by current speakers. The window of opportunity for OIRs is thus larger than for other next turns, which are subject to constraints on turn allocation and overlap that OIRs appear to out rank.

This is not to say that the timing of OIR is without constraint. The organization of repair imposes certain constraints on the timing of OIRs vis-à-vis the selection of OIR practices (see Robinson, 2014). An open OIR locates a trouble-source TCU exclusively via adjacency and is therefore positionally restricted. If a next speaker fails to hear or understand a TCU well enough to employ a specific OIR practice, then the window of opportunity to use an open OIR has an outer bound: the possible completion of a subsequent TCU that intervenes between the trouble-source TCU and the open OIR. In other words, the opportunity space for open OIR is a one-TCU interval (Robinson, 2014). But does *this* constraint, which operates for open OIRs, establish a motivation for fast turn transitions? It provides an outer bound for the timing of open OIRs, but given that an open OIR can in principle occur in overlap with a subsequent TCU (before its possible completion) and take priority, it would not systematically motivate a fast transition on the order of 100–300 ms between the trouble-source TCU and the OIR.

What, then, of the potential for silences to be meaningful, as signals of interactional trouble? Might this provide a motivation for fast transitions? The answer becomes clear once one registers that OIRs are themselves signals of interactional trouble. Although a covert signal of trouble like silence will be at cross-purposes with some incipient actions (e.g., agreement), it can also point in the same direction as an incipient action (e.g., rejection), in which case it is interactionally advantageous. An expanded transition space can indicate that a next speaker has encountered trouble, but it also provides an opportunity for the resolution of the trouble. There is reason to believe that speakers do not *avoid* this covert signal (e.g., by initiating repair quickly), but rather *exploit* it. As a motivation for fast turn transfers, the potential for silences in conversation to be meaningful signals of interactional trouble is thus context-sensitive, operating only for next turns not themselves designed as signals of trouble.

In sum, the rules and constraints of the turn-taking system that motivate fast transitions – concerning overlap management, turn allocation, and the semiotics of silence – neither rule nor constrain the timing of OIRs. The priority given, at the intersection of turn-taking and repair, to the resolution of troubles in hearing and understanding provides a systemic explanation for the observation that transitions before OIRs tend to be longer than those before other next turns, for which the rules and constraints of the turn-taking system remain operative.

## Conflict of Interest Statement

The author declares that the research was conducted in the absence of any commercial or financial relationships that could be construed as a potential conflict of interest.

## Footnotes

1 An examination of visual signals that precede open OIRs – which have the longest transition spaces on average and are therefore the most pertinent to the question – revealed relevant visible bodily actions such as those described in this section in 26.4% of cases (*n =*14). However, the corpus is not well suited to a quantitative analysis of visible bodily actions. In some cases, the relevant participant is off-camera or his or her face cannot be seen due to the angle of the camera, and in many cases, the participant’s eyes cannot be seen well enough to measure his or her gaze. A corpus specifically designed for the temporal analysis of gaze and gesture (e.g., Holler and Kendrick, 2015) is needed to analyze the visual signals that precede OIRs quantitatively.
